# Elevation of the antifibrotic peptide *N*-acetyl-seryl-aspartyl-lysyl-proline: a blood pressure-independent beneficial effect of angiotensin I-converting enzyme inhibitors

**DOI:** 10.1186/1755-1536-4-25

**Published:** 2011-11-30

**Authors:** Megumi Kanasaki, Takako Nagai, Munehiro Kitada, Daisuke Koya, Keizo Kanasaki

**Affiliations:** 1 Division of Diabetes & Endocrinology, Kanazawa Medical University, Uchinada, Ishikawa, Japan

## Abstract

Blockade of the renin-angiotensin system (RAS) is well recognized as an essential therapy in hypertensive, heart, and kidney diseases. There are several classes of drugs that block the RAS; these drugs are known to exhibit antifibrotic action. An analysis of the molecular mechanisms of action for these drugs can reveal potential differences in their antifibrotic roles. In this review, we discuss the antifibrotic action of RAS blockade with an emphasis on the potential importance of angiotensin I-converting enzyme (ACE) inhibition associated with the antifibrotic peptide *N*-acetyl-seryl-aspartyl-lysyl-proline (AcSDKP).

## Introduction

In recent decades there has been a tremendous increase in the therapeutic options available for the inhibition of the renin-angiotensin system (RAS). Historically, angiotensin-converting enzyme inhibitors (ACE-I) were the first class of RAS inhibitors identified. The first ACE-I, captopril, was discovered by a scientist at Squibb, a US pharmaceutical company, in 1975 [1]. Captopril was based on the peptide sequence of bradykinin-potentiating factor, which inhibited the conversion of angiotensin I to angiotensin II when perfused into pulmonary circulation [2].

Approximately 15 years ago, a second class of RAS inhibitors was introduced into the market, the angiotensin II receptor blockers (ARBs) [3]. Very recently, a novel class of RAS inhibitor, including aliskiren [4], which directly inhibits renin has been put into clinical use. Most of the literature support the beneficial effects of this novel class of RAS inhibitors as antihypertensive drugs [5,6]. Interestingly, the use of these drugs is not limited to antihypertensive disorders. The clinical use of RAS inhibitors has emerged as beneficial for the prevention of diabetes [7,8], fibrotic kidney disease [9], heart disease [10], aging [11] and Alzheimer's disease [12].

There is no doubt that RAS inhibitors are beneficial drugs; however, the differences between each of these classes of inhibitors are not yet clear. After a brief introduction to the RAS, we analyze the potential differences between ACE-I and ARBs as antifibrotic drugs. Emphasis is placed on the ACE inhibitors and the antifibrotic peptide AcSDKP.

### RAS

Renin, an aspartyl protease, was discovered by Robert Tigerstedt at the Karolinska Institute in 1898 [13]. The majority of renin in the body is found in the juxtaglomerular cells of the kidney. Additionally, renin has been found in many other tissues but without clear mechanistic evidence of its function in these locations [14]. Renin cleaves angiotensinogen, which results in the production of the decapeptide angiotensin I. The octapeptide angiotensin II, a potent vasoconstrictor, is formed by ACE-mediated cleavage of angiotensin I.

**Figure 1:**
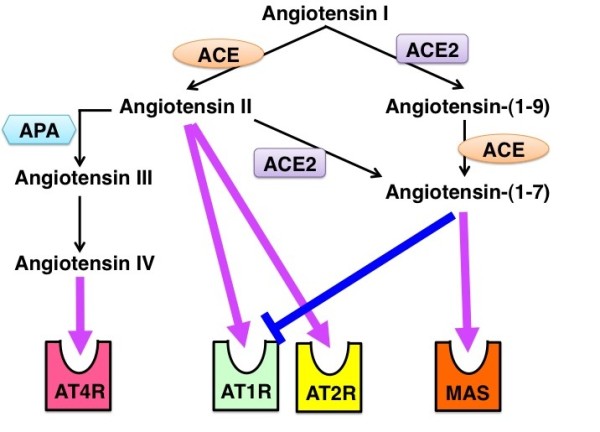
**Overview of angiotensin-converting enzyme (ACE)/ACE2 action and synthesis of bioactive angiotensin peptides**. ACE metabolizes angiotensin I into angiotensin II. Angiotensin II is cleaved by aminopeptidase A (APA) into angiotensin III and subsequently angiotensin IV. Angiotensin I is also cleaved by ACE2 into angiotensin-(1-9). Angiotensin-(1-7) is synthesized from angiotensin-(1-9) by ACE or alternatively from angiotensin II by ACE2. These angiotensin peptides bind to specific receptors and exhibit biological functions.

There are two main receptors for angiotensin II (AT1 and AT2), which are differentially expressed on the cell surface (Figure 1) [15]. Those receptors share the configuration of a seven-transmembrane receptor but exhibit only around 20% protein sequence homology [16]. These two receptors play distinct physiological roles [16]. AT1 receptors are coupled to G proteins and mediate diverse signaling pathways, such as activation of phospholipases, inhibition of adenylate cyclase, and stimulation of tyrosine phosphorylation [15]. However, the interaction of AT2 receptors and G proteins is controversial [17]. These two receptors are differentially regulated during the development [18]. When analyzed in lamb, AT2 receptors are expressed abundantly in the fetal kidney, especially in the undifferentiated mesenchyme [18]. These AT2 receptors are at decreased expression levels after birth [18]. AT1 receptors are initially expressed in the nephrogenic cortex and developing glomeruli, proximal tubule and vessels; they become more abundant through the development processes [18].

The expression of AT1 receptors is stimulated by several conditions, such as high cholesterol levels and osmolarity changes, but decreased by high concentration of angiotensin II [15]. Such angiotensin II-dependent downregulation is not found for AT2 receptors; instead, AT2 receptors are induced by tissue injury [17]. Indeed, AT2 receptors are re-expressed by renal injury and the nephron remodeling processes [17].

Vasoconstriction, profibrotic action, growth stimulation, aldosterone release and proinflammatory functions are classical angiotensin II-driven physiological functions that are mediated by AT1 receptors [19]. AT2 receptor-mediated signaling may antagonize AT1-mediated signal transductions [20-22]. However, accumulating evidence indicates that AT2 receptor-mediated signaling also mediates the detrimental action of angiotensin II, including hypertrophy [23,24], and the stimulation of proinflammatory pathway nuclear factor κB [25,26]. In this regard, blockade of the AT2 receptor by a specific inhibitor was associated with the inhibition of inflammation and renoprotection in subtotally nephrectomized rats [27].

In addition to classical members, some new bioactive molecules, such as angiotensin IV and angiotensin-(1-7), have been introduced in RAS systems.

Angiotensin II is metabolized by aminopeptidase A (APA) into antgiotensin III and finally angiotensin IV (Figure 1) [28]. Angiotensin IV binds to the specific receptor AT4 (Figure 1), which is reported to be an insulin-regulated membrane aminopeptidase [29,30]. It is reported that angiotensin IV can induce plasminogen activator inhibitor (PAI)-1 expression in the proximal tubule and vascular endothelial cells [29,31]. PAI-1 activation has been associated with the reduction of extracellular matrix turnover [32]; angiotensin IV-mediated signaling may be associated with the tissue fibrosis [31]. The angiotensin IV-generating enzyme APA is induced in conditions of renal injury and high angiotensin II levels [28]; subsequently, more angiotensin II is utilized in the production of angiotensin IV. Angiotensin IV is also associated with the release of nitric oxide and focal adhesion kinase phosphorylations [33,34]. Interestingly, the angiotensin IV/AT4 receptor signaling pathway has been shown to be involved in glucose homeostasis [35,36] and cognitive functions [37], suggesting diverse physiological roles of this pathway.

Another RAS-derived bioactive molecule is angiotensin-(1-7), which has been shown to inhibit the effects of angiotensin II (Figure 1) [38]. For example, angiotensin-(1-7) plays a role as an antihypertensive molecule through the stimulation of the release of vasodilator prostaglandins and nitric oxides [38]. In addition to such antihypertensive effects, angiotensin-(1-7) inhibits the angiotensin II-induced proliferation and growth stimulation signal in vascular smooth muscle cells [39-41]. Most likely, these effects of angiotensin-(1-7) as a negative regulator of angiotensin II are mediated, at least in part, by the downregulation of the angiotensin II receptor AT1 (Figure 1) [42]. Also, it is reported that angiotensin-(1-7) is the endogenous ligand for the MAS receptor (Figure 1) [43]. Studies utilizing MAS receptor deficient mice have indicated that the interaction between angiotensin-(1-7) and the MAS receptor plays vital roles in heart function [43], sympathetic tone regulation [44], aortic relaxation [45], and endothelial function [46].

The synthesis of angiotensin-(1-7) is mediated by a unique RAS pathway involving ACE2 (Figure 1) [47,48]. ACE2 is expressed predominantly in vascular endothelial cells of the heart and kidney [47,49]. Both ACE and ACE2 metabolize angiotensin I. However, the resulting peptides are different (Figure 1). As shown above, ACE converts angiotensin I to the octapeptide angiotensin II, whereas ACE2 cleaves one amino acid from angiotensin I; subsequently, nonapeptide angiotensin 1-9 is synthesized (Figure 1) [47]. Although angiotensin 1-9 itself exhibits no known biologic activity, angiotensin 1-9 is cleaved by ACE, and bioactive angiotensin-(1-7) is synthesized (Figure 1) [50]. ACE2 can also directly cleave angiotensin II to form angiotensin-(1-7) (Figure 1); therefore, this angiotensin II degradation product exhibits properties that are opposite those of angiotensin II [48].

### RAS activation and tissue fibrosis

Activation of RAS and production of angiotensin II is associated with tissue fibrosis [51,52]. Angiotensin II stimulates extracellular matrix accumulation and collagen deposition through the induction of mitogen activated protein kinases (MAPKs), such as extracellular signal-regulated kinase (ERK) [53], p38 [54] and c-Jun N-terminal kinases (JNKs) [55], *in vivo *and *in vitro*. Additionally, angiotensin II stimulates the expression of the profibrotic cytokine transforming growth factor (TGF)β in rat cadiac fibroblasts [56] and connective tissue growth factor in rat tubular epithelial cells [57]. Some reports have also indicated that angiotensin II may directly activate Smad proteins, which are part of the intracellular TGFβ signaling pathway [58-60]. Furthermore, angiotensin II stimulates rat cardiac fibroblast proliferation [61]. In addition to the angiotensin II/AT1 receptor-mediated major profibrotic signaling pathways in RAS, the angiotensin IV/AT4 receptor pathway could contribute to tissue fibrosis via the induction of PAI-1 [31], as described above.

Therefore, appropriate inhibition of profibrotic angiotensins such as angiotensin II or angiotensin IV, production pathways, or, alternatively, activation of an antiprofibrotic angiotensin pathway such as ACE2 or angiotensin-(1-7) could be a potential route for antifibrotic therapy. In this regard, currently available RAS inhibitors, such as ACE-I and ARB, are somewhat reasonable as antifibrotic drugs. However, there are differences in the antifibrotic molecular mechanisms of these drugs.

### ACE inhibitors

ACE inhibitors are members of the first class of RAS inhibitors. The first ACE inhibitor to be used in the clinic, captopril, showed dramatic beneficial effects in type I diabetic patients with nephropathy [62]. Following this study, several clinical trials demonstrated that ACE inhibition could significantly prevent the progression of renal disease [63-65].

The ACE-I class of drugs exhibit their antihypertensive effects by inhibiting the conversion of angiotensin II from angiotensin I. ACE-I inhibition has been shown to decrease fibrosis in experimental models of heart [66] and kidney [67-69] disease. Because angiotensin II is a profibrogenic molecule, it would be logical to conclude that the beneficial effects of ACE-I are mediated through the inhibition of angiotensin II production; however, the beneficial effects of ACE-I cannot be explained by the suppression of angiotensin II production alone because maximal doses of ACE-I may not be sufficient to inhibit all the biosynthesis of angiotensin II [16]. Indeed, systemic administration of ACE-I has little effect on the formation of angiotensin II in the kidney, even though such ACE-I can almost completely inhibit systemic angiotensin II formation from angiotensin I [70]. Therefore, it is likely that the decrease in the production of angiotensin II is not the only mechanism underlying the antifibrotic effects of ACE-I.

### ACE inhibition and elevation of the antifibrotic peptide *N*-acetyl-seryl-aspartyl-lysyl-proline (AcSDKP)

AcSDKP is a tetrapeptide originally isolated from fetal calf bone marrow [71], and has recently emerged as an antifibrosis molecule.

**Figure 2:**
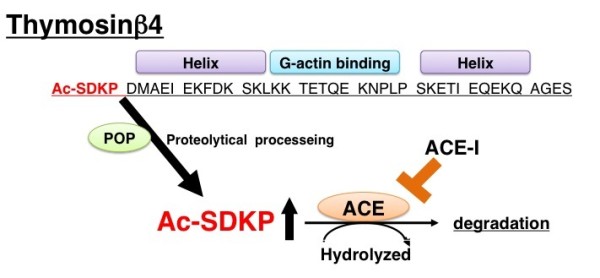
**Amino-acid sequence of thymosin β4 and endogenous formation of *N*-acetyl-seryl-aspartyl-lysyl-proline (AcSDKP)**. G actin binding peptide thymosin β4 is cleaved by an endopeptidase, likely prolyl oligopeptidase (POP), and subsequently its N-terminal tetrapeptide AcSDKP is synthesized. AcSDKP is hydrolyzed and degraded by angiotensin-converting enzyme (ACE). Therefore, when ACE inhibitors are used, the concentration of AcSDKP increases.

Details of the endogenous synthesis of AcSDKP are not yet clear; however, available information strongly suggests that thymosin β4 (Tβ4) is the most likely candidate precursor of AcSDKP [72,73] (Figure 2). Lenfan *et al*. showed that incubation of [^3^H] Tβ4 with bone marrow cells or bone marrow lysate resulted in the formation of [^3^H]AcSDKP [72]. Furthermore, Tβ4 knockdown utilizing the small interfering (si)RNA for Tβ4 led to significant reduction of AcSDKP in HeLa cells [73]. AcSDKP is the N-terminal sequence of Tβ4 (Figure 2). AcSDKP was believed to be synthesized by a single cleavage employing Asp-N endopeptidase [72]. However, Asp-N was only found in bacteria; therefore, Cavasin *et al*. tried to find another enzyme responsible for the synthesis of AcSDKP from Tβ4 [74]. Subsequently, they found that prolyl oligopeptidase (POP) is responsible for the formation of AcSDKP and that POP inhibitors blocked the formation of AcSDKP from Tβ4 [74] (Figure 2).

Tβ4 is a ubiquitously distributed 43-amino-acid peptide (4.9 kDa), originally identified as an intracellular peptide, which can sequester G-actin and regulate its polymerization [75,76]. In addition to the role as actin polymerizations, Tβ4 exhibits various biologically significant activities [75,76]. Interestingly, Bock-Marquette *et al*. reported that the administration of exogenous intracardiac and intraperitoneal Tβ4 significantly restored cardiac functions associated with neovascularization in an experimental myocardial infarction model of mice [77] and epicardial progenitor mobilization [78], suggesting that Tβ4 exhibit extracellular organ-protective roles associated with antifibrosis and enhanced angiogenesis.

AcSDKP is a natural inhibitor of hematopoietic stem cell proliferation that prevents entry into S phase from G1 in the cell cycle [79]. Interestingly, AcSDKP is hydrolyzed in the presence of ACE (Figure 2). Therefore, plasma levels of AcSDKP are minimal in normal conditions, whereas ACE-I administration leads to a fivefold increase in its concentration [80]. AcSDKP has been shown to suppress the proliferation of human mesangial cells [81] and renal fibroblasts [82], in addition to inhibiting collagen deposition in mouse cardiac fibroblasts [83]. The administration of AcSDKP ameliorated renal fibrosis and glomerular sclerosis in hypertensive rat models and diabetic and non-diabetic kidney disease models without altering blood pressure [84,85]. These observations suggest that the renoprotective effects of ACE-I are mediated, at least in part, by the accumulation of AcSDKP (Figure 2).

**Figure 3:**
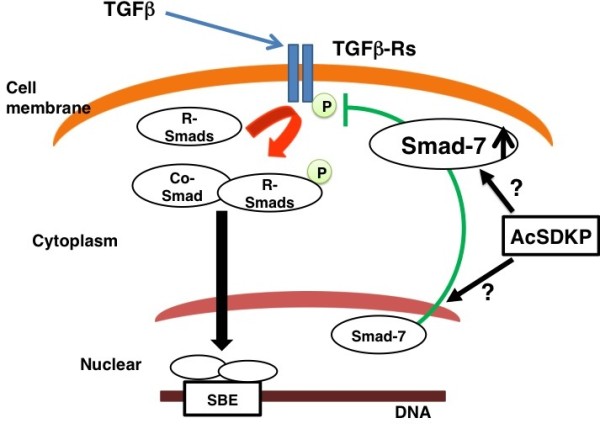
**The action of *N*-acetyl-seryl-aspartyl-lysyl-proline (AcSDKP) on transforming growth factor (TGF)β signal transduction**. TGFβ binds to TGF receptors on the cell membrane. TGFβ and TGFβ-receptor interaction induces phosphorylation of receptor-regulated (R)-Smads. Phosphorylated R-Smads bind with the common (co)-Smad in the cytoplasm of cells. Such Smads heterodimerize in the nucleus and bind to the genomic promoter region of DNA, called the Smad binding element (SBE). AcSDKP may induce Smad7 translocation from the nucleus of cells to the cytoplasm and inhibit phosphorylation of R-Smads by receptors. Or alternatively, AcSDKP may increase Smad7 levels in the cytoplasm by as-yet unknown mechanisms.

Our group and others have shown that AcSDKP prevents Smad2 phosphorylation (Figure 3) and that this molecular mechanism may mediate its antifibrotic effect [86,87]. This observation identifies AcSDKP as the first circulating, endogenous inhibitor of Smad2 phosphorylation.

The Smads are transcription factors specific to the TGFβ family, and they play essential roles in signal transduction from the cell membrane [88,89]. Smads are classified into three types: (1) receptor-regulated Smads, or R-Smads (Smad2 and 3); (2) common Smad, or co-Smad (Smad4); and (3) inhibitory Smads, or I-Smads (Smad6 and 7). Upon TGFβ binding, the type II receptor interacts with the type I receptor, which induces phosphorylation of serine residues in the type I receptor (Figure 3) [90]. Subsequently, the phosphorylated type I receptor recruits R-Smads to be phosphorylated, and phosphorylated R-Smads interact with co-Smad in the cytoplasm of cells (Figure 3). This R-Smad and co-Smad heterodimer is imported into the nucleus (Figure 3) with the help of importin-β [91,92]. The Smad heterodimer binds to Smad-binding elements in the promoter regions of DNA (Figure 3). Under normal conditions, I-Smad is localized to the nucleus (Figure 3) [87]. E3 ubiquitin ligase Smurfs mediate the translocation of nuclear-localized I-Smads to the cytoplasm following TGFβ stimulation. Cytoplasmic I-Smad competitively inhibits R-Smad phosphorylation by the type I receptor (Figure 3) [93]. Ubiquitination of receptors by I-Smad-associated Smurfs are also part of the negative feedback loop between TGFβ and the I-Smads [94-96].

How does AcSDKP inhibit TGFβ-induced phosphorylation of R-Smad? This effect is likely associated with the activation of I-Smads (Figure 3). Incubation of human mesangial cells in the presence of AcSDKP leads to cytoplasmic mobilization of Smad7, one of the I-Smads, in the absence of TGFβ stimulation (Figure 3) [87]. Our group and others have reported increased Smad7 levels *in vivo *following AcSDKP administration, supporting this Smad7-mediated anti-TGFβ effect by AcSDKP (Figure 3) [97,98]. Additional information related to the mechanism underlying the AcSDKP-mediated translocation and increase in Smad7 concentration is not clear. Interestingly, AcSDKP also inhibits cell cycle progression stimulated by serum-derived or platelet-derived growth factor-B in human mesangial cells by inhibiting the degradation of p53, p27^kip1 ^and p21^cip1 ^[81]. Similar to Smad7 [96], these molecules are exclusively degraded by the ubiquitin-proteasome pathway [99]; therefore, it is possible that AcSDKP may inhibit the Smad7 degradation pathway.

**Figure 4:**
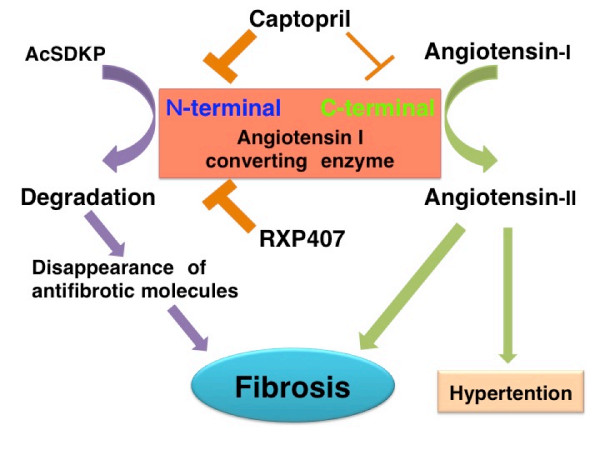
**The biology of angiotensin-converting enzyme (ACE) in tissue fibrosis**. Angiotensin-converting enzyme has two catalytic sites. Angiotensin I exhibits higher affinity for the C-terminal catalytic site of ACE. Degradation of the antifibrotic molecule *N*-acetyl-seryl-aspartyl-lysyl-proline (AcSDKP) is exclusively induced at its N-terminal catalytic site. Therefore ACE induces tissue fibrosis by both the production of angiotensin II and the decreased level of AcSDKP. The ACE inhibitor captopril displays higher affinity for the N-terminal catalytic sites of ACE when compared to C-terminal catalytic sites. RXP407, a specific inhibitor for the ACE N-terminal catalytic site, may increase concentrations of AcSDKP and exhibit an antifibrotic action.

ACE has N-terminal and C-terminal catalytic domains responsible for interactions with and cleavage of target substrates (Figure 4) [100]. Evidence suggests that these two catalytic domains may be different (Figure 4). Bradykinin is hydrolyzed at approximately the same rate by both of these catalytic sites. Although angiotensin I can be cleaved by either catalytic domain, the C-terminal domain has a threefold higher affinity for angiotensin I (Figure 4) [100,101]. Interestingly, AcSDKP is hydrolyzed exclusively by the N-terminal catalytic domain (Figure 4) [102]. Importantly, each ACE-I exhibits a distinct affinity for each of the catalytic domains; for example, captopril displays a higher affinity for the N-terminal catalytic domain (Figure 4). It is likely that the hydrophobic moieties of ACE-I play an essential role in this domain selectivity [103]. It was recently reported by Li *et al*. that mice deficient for the N-terminal catalytic domain of ACE exhibited an antifibrotic effect due to an accumulation of AcSDKP, revealing the importance of the N-terminal domain for the antifibrotic actions of ACE-I (Figure 4) [104]. In addition to the full-length somatic form of ACE, there is a transcriptional variant with an N-terminal deletion, known as the germinal form [100]. The testes, which express germinal-type ACE, are associated with higher levels of AcSDKP relative to other tissues [105,106]. AcSDKP and its precursor peptide, Tβ4, were able to rescue fibrotic heart disease in a preclinical model [78]. These results demonstrated the importance of the antifibrotic effect of AcSDKP in the inhibition of ACE. Therefore, the N-terminal catalytic domain-specific inhibitor of ACE, RXP407 (Figure 4), has great potential as an antifibrotic therapy [107-110].

### Angiotensin type I receptor blocker vs ACE inhibition

There may be potential problems with the long-term clinical use of ACE-I to inhibit the RAS. The prolonged use of ACE-I leads to the compensatory upregulation of angiotensin I [111]. Under these conditions, known as aldosterone escape, chymase may act as the converting enzyme to generate angiotensin II [111]. Therefore, a strategy that prevents angiotensin II from binding to the angiotensin type I receptor is necessary. To address this problem, ARBs, such as losartan, were developed as a novel class of RAS inhibitors [3]. Large clinical trials, such as the RENAAL study, have revealed that losartan exhibits renoprotective effects and inhibits overall mortality in type 2 diabetic nephropathy patients with overt proteinuria [112]. Other studies have also reported similar renoprotective effects associated with an increase in overall mortality. The clinical use of ARB is much easier than that of ACE-I given the side effects typically associated with the latter, such as dry cough, which leads to poor compliance in patients prescribed the drug. Because ARBs inhibit only the AT1 signaling pathway, they were thought of as an ideal strategy to treat hypertensive patients with kidney diseases.

As pharmacological function and effector target are different, ACE-I and ARB exhibit different influences in RAS-dependent and RAS-independent pathways, such as the AcSDKP accumulation by ACE-I described above.

ACE inhibition by ACE-I leads to a suppression of angiotensin II formation, resulting in less angiotensin II binding to the AT1 receptor as well as the AT2 receptor [16]. However, when an ARB is utilized, AT1 receptor signaling is inhibited; angiotensin II accumulates, and subsequently, such increased angiotensin II binds and activates AT2 receptors [22]. As shown above, stimulation of the AT2 receptor may be detrimental for organ protection (and may also antagonize the AT1 receptor-mediated profibrotic signal in some experimental conditions) [22].

Even though ACE-I may not directly suppress ACE2, ACE-I might inhibit the formation of antihypertensive/antifibrotic angiotensin-(1-7) in an indirect fashion, because conversion of angiotensin 1-9 to angiotensin-(1-7) is mediated by ACE [50]. For angiotensin-(1-7), ARB may increase its formation via accumulated angiotensin II directly cleaved by ACE2 [50].

Another difference between ACE-I and ARB is the concentration of plasma bradykinin [113]. Bradykinin breakdown is mediated by ACE; therefore, ACE-I treatment increases bradykinin concentration [113]. Using bradykinin B2 receptor knockout mice, Schanstra *et al*. reported that the bradykinin B2 receptor signaling pathway exhibited antifibrotic roles associated with the induction of plasminogen activators/matrix metalloproteinase-2, enzymes associated with extracellular matrix degradation in the unilateral ureteral obstruction (UUO) model of renal fibrosis [114]. Moreover, Akita diabetic mice lacking the bradykinin B2 receptor developed overt nephropathy when compared to control mice [115]. However, the role of ACE inhibition and bradykinin B2 signaling pathway activation is still controversial because it was also shown that ACE-I treatment in the UUO model using either bradykinin B2 receptor knockout mice or control mice demonstrated that ACE-I exhibited a significant reduction in renal fibrosis in all groups [116], suggesting that the presence of bradykinin B2 receptor signaling may not be necessary for the tissue protection mediated by ACE-I in this model [116]. Nevertheless, cell biology analysis in human mesangial cells revealed that bradykinin and the bradykinin B2 receptor pathway might contribute to the therapeutic effect of the ACE-I inhibitor perindoprilat during mesangial scarring [117].

ACE-I and ARB combination therapy likely show additive antihypertensive and organ protective effects because these two therapies exhibit diverse RAS-dependent and RAS-independent pathway activity [118-123]; however, some trials have shown that combination therapy may not be renoprotective, despite the significant reduction in proteinuria levels [124]. This discrepancy between the trials could be dependent upon the specific drug used and the design of the trials [118-124]. Mauer *et al*. recently reported on important differences between ACE-I and ARBs [125]. They found that ARBs enhanced progression of microalbuminuria in early type I diabetes patients with normotensive and normoalbuminuria. Such enhanced progression of microalbuminuria is associated with a trend of increased mesangial fractional volume in glomeruli in the kidney [125]. Furthermore, treatment with ACE-I showed no significant differences between patients in the treatment or control groups [125]. The conundrum of this study is that the onset of diabetic retinopathy was inhibited by either ARB or ACE-I treatment, suggesting that both drugs efficiently inhibited angiotensin II stimulated signaling pathway in both groups [125]. A follow-up study is clearly necessary to clarify the therapeutic approach for early diabetes patients to prevent the onset of more advanced kidney disease.

One possible explanation for these unexpected results is that the angiotensin II-mediated signaling pathway may not contribute to the onset of microalbuminuria and mesangial matrix accumulation of the kidney in early diabetic normotensive patients with normoalbuminuria. Another possibility is that the administration of ARBs results in local ACE activation [16,126]; in other words, despite blocking the angiotensin II receptor signaling pathway, activation of an angiotensin-independent, profibrotic pathway mediated by ACE may occur, such as the accelerated degradation of AcSDKP (Figure 4). ACE-I, but not ARBs, inhibited murine adriamycin nephropathy, suggesting that diverse pathways may be involved in fibrotic diseases [68].

### Anti-inflammatory, antiapoptotic and proangiogenic roles of AcSDKP

**Figure 5:**
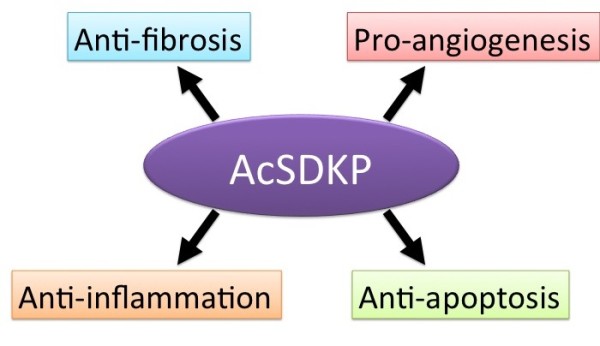
***N*-acetyl-seryl-aspartyl-lysyl-proline (AcSDKP) as an attractive target molecule for fighting tissue fibrosis**. AcSDKP exhibits multiple functions, such as antifibrosis, anti-inflammation, antiapoptosis and proangiogenesis, and could be a candidate target molecule for novel antifibrotic drugs.

Because AcSDKP was originally identified as a hematopoietic stem cell regulator [71,127,128], there have been many studies performed utilizing bone marrow cells. AcSDKP inhibits apoptosis (Figure 5) induced by cytotoxic stresses, including chemotherapy [129,130], radiation [131,132], high temperature [133-135] and photofrin II-mediated phototherapy [136]. Increased apoptosis is associated with tissue fibrosis, and its inhibition has been linked to the restoration of fibrosis in several organs [137-140].

Inflammation is also associated with tissue fibrosis [137-140]. In experimental animal models, AcSDKP inhibited inflammation in the kidney, heart and liver that was associated with the amelioration of tissue fibrosis (Figure 5) [97,98,141-145]. Although the precise molecular mechanisms explaining how AcSDKP inhibits inflammation are not yet clear, it is likely that the suppression of MCP-1 contributes to these anti-inflammatory effects [146]. Inhibition of MCP-1 is likely associated with the inhibition of MAPK activation *in vivo *[147]. However, the effects of AcSDKP on MAPK are cell-type dependent, as suggested by the fact that AcSDKP both inhibits and stimulates ERK phosphorylation in different cellular contexts, such as in rat cardiac fibroblasts [83,148] and human mesangial cells, respectively [81,87]. This finding suggests that AcSDKP is not simply a MAPK inhibitor.

Angiogenesis is important in protection from tissue damage and the promotion of tissue repair. Interestingly, both AcSDKP (Figure 5) [149-151] and its precursor peptide, Tβ4 [75,152-154], enhanced angiogenesis and exhibited antifibrotic effects associated with the normalization of organ function [78]. AcSDKP has been shown to improve skin flap survival and accelerate wound healing [151]. The relationship between tumor angiogenesis and Tβ4 with AcSDKP has been extensively studied by Wdzieczak-Bakala's research group. They have proposed that high levels of Tβ4 and AcSDKP are associated with tumor progression in hematologic malignancies [73,155-157]. Angiogenesis plays a pivotal role in cancer development [158,159], and AcSDKP levels are markedly elevated in both hematologic malignancies and solid neoplasms [73,155-157]. An association between the levels of AcSDKP and tumor angiogenesis was observed in these studies; however, the pathophysiological significance of this result has not been clearly shown.

Finally, AcSDKP infusion inhibited liver injury associated with the inhibition of the TGFβ and Smad pathways in carbon tetrachloride-induced liver fibrosis models [141]. Additionally, this treatment was associated with the induction of bone-morphogenetic protein 7 (BMP-7) [141], a promising antifibrotic molecule [160,161]. The antifibrotic, antiapoptotic, anti-inflammatory and proangiogenic properties of BMP-7 have been well established [160,161]. Therefore, it is possible that AcSDKP could function, in part, through the induction of BMP-7 [141]. Furthermore, this same study demonstrated that AcSDKP induced expression of the potent anti-inflammatory transcription factor peroxisome proliferator-activated receptor (PPAR)-γ [141].  PPAR-γ is not only a potent anti-inflammatory transcription factor, but it is also a critical regulator for adipogenesis, lipogenesis and insulin sensitivity [141].

### Perspective

We have summarized the potential beneficial effects of AcSDKP in fibrotic diseases. It is obvious that the antifibrotic effect of AcSDKP is associated with its anti-inflammatory, antiapoptotic and proangiogenic properties (Figure 5). Therefore, AcSDKP appears to be an attractive molecule for antifibrotic therapy. One of the problems associated with the use of this molecule as an antifibrotic therapy is its short half-life of approximately 5 min in plasma [162,163]. It would be possible to make an AcSDKP analogue with an extended half-life; however, the hematopoietic effects of AcSDKP are diminished when single amino-acid modifications are made [164]. Therefore, the best approach for converting AcSDKP into a practical antifibrotic agent would be to manufacture a small molecule that mimics AcSDKP function for oral intake. Alternatively, inactivation of the N-terminal catalytic function of ACE could be used as a therapeutic approach; however, some critical information is missing to make this approach feasible. First, the direct target of AcSDKP and its exact function are not clear, even though AcSDKP is known to induce the accumulation of Smad7 and various cell cycle modulators responsible for inhibiting the TGFβ/Smad signaling pathway and inducing the antiproliferative effects on fibroblasts and mesangial cells, respectively [81,87,97,98]. Second, it is not known whether AcSDKP acts as a ligand for its own receptor or if there are any receptors for AcSDKP [165]. Alternatively, AcSDKP may enter cells by phagocytosis and inhibit intracellular signaling pathways. Third, specific inactivation of the N-terminal catalytic domain of ACE by an inhibitor, such as RX407, can be used in the clinic to induce AcSDKP without the side effects associated with conventional ACE-I, such as dry cough [108-110]. Such information is essential if we hope to develop novel antifibrotic therapies based on enhancing the function of AcSDKP. Additionally, the significance of physiological changes in AcSDKP levels must be analyzed in human patients with fibrotic diseases.

## Conclusions

Tissue fibrosis is associated with organ damage and dysfunction, which are the major causes of disability and death in these patients. Specific therapies to treat fibrosis are not yet available in the clinic. Although tissue fibrosis is detrimental to organ function, it may also be a component of homeostasis and repair pathways. Therefore, caution should be used to determine whether AcSDKP is harmful in a subset of patients. We must carefully consider the potential therapeutic utilization of AcSDKP and its role in other diseases [73,155-157]. Clearly, more research is needed into the regulation of AcSDKP levels to show its effectiveness and safety as a therapeutic agent. Nonetheless, AcSDKP remains an attractive target as a potential antifibrotic strategy.

## Competing interests

The authors declare that they have no competing interests.

## Authors' contributions

MK contributed to writing the manuscript. TN made the figures. MK was involved in the discussion. DK made intellectual contributions. KK conceived the project, provided intellectual contributions, and contributed to the manuscript writing and editing. All authors read and approved the final manuscript.

## References

[B1] Cushman DW Ondetti MA History of the design of captopril and related inhibitors of angiotensin converting enzyme Hypertension 1991 17 589 592 10.1161/01.hyp.17.4.589 2013486

[B2] Smith CG Vane JR The discovery of captopril FASEB J 2003 17 788 789 10.1096/fj.03-0093life 12724335

[B3] Aulakh GK Sodhi RK Singh M An update on non-peptide angiotensin receptor antagonists and related RAAS modulators Life Sci 2007 81 615 639 10.1016/j.lfs.2007.06.007 17692338

[B4] Wood JM Maibaum J Rahuel J Grütter MG Cohen NC Rasetti V Rüger H Göschke R Stutz S Fuhrer W Schilling W Rigollier P Yamaguchi Y Cumin F Baum HP Schnell CR Herold P Mah R Jensen C O'Brien E Stanton A Bedigian MP Structure-based design of aliskiren, a novel orally effective renin inhibitor Biochem Biophys Res Commun 2003 308 698 705 10.1016/s0006-291x(03)01451-7 12927775

[B5] Parving HH Persson F Lewis JB Lewis EJ Hollenberg NK Aliskiren combined with losartan in type 2 diabetes and nephropathy New Engl J Med 2008 358 2433 2446 10.1056/NEJMoa0708379 18525041

[B6] Solomon SD Appelbaum E Manning WJ Verma A Berglund T Lukashevich V Cherif Papst C Smith BA Dahlof B Effect of the direct Renin inhibitor aliskiren, the Angiotensin receptor blocker losartan, or both on left ventricular mass in patients with hypertension and left ventricular hypertrophy Circulation 2009 119 530 537 10.1161/CIRCULATIONAHA.108.826214 19153265

[B7] Padwal R Laupacis A Antihypertensive therapy and incidence of type 2 diabetes: a systematic review Diabetes Care 2004 27 247 255 10.2337/diacare.27.1.247 14693997

[B8] Scheen AJ Renin-angiotensin system inhibition prevents type 2 diabetes mellitus. Part 1. A meta-analysis of randomised clinical trials Diabetes Metabol 2004 30 487 496 10.1016/s1262-3636(07)70146-5 15671918

[B9] Scheen AJ Prevention of type 2 diabetes mellitus through inhibition of the Renin-Angiotensin system Drugs 2004 64 2537 2565 10.2165/00003495-200464220-00004 15516153

[B10] Deelman L Sharma K Mechanisms of kidney fibrosis and the role of antifibrotic therapies Curr Opin Nephrol Hypertens 2009 18 85 90 10.1097/MNH.0b013e32831c50a1 19077695

[B11] de Cavanagh EM Inserra F Ferder L Angiotensin II blockade: a strategy to slow ageing by protecting mitochondria? Cardiovasc Res 2011 89 31 40 10.1093/cvr/cvq285 20819950

[B12] Phillips MI de Oliveira EM Brain renin angiotensin in disease J Mol Med 2008 86 715 722 10.1007/s00109-008-0331-5 PMC7095973 18385968

[B13] Tigerstedt R Bergman P Niere und kreislauf Scand Arch Physio 1898 8 223 271

[B14] Campbell DJ Extrarenal renin and blood pressure regulation. An alternative viewpoint Am J Hypertens 1989 2 266 275 10.1093/ajh/2.4.266 2650709

[B15] Bernstein KE Sayeski PP Doan T Farmer PK Ali MS Signal transduction pathways of angiotensin II in the kidney Contrib Nephrol 2001 135 16 33 10.1159/000060168 11705281

[B16] Wolf G Ritz E Combination therapy with ACE inhibitors and angiotensin II receptor blockers to halt progression of chronic renal disease: pathophysiology and indications Kidney Int 2005 67 799 812 10.1111/j.1523-1755.2005.00145.x 15698420

[B17] Gallinat S Busche S Raizada MK Sumners C The angiotensin II type 2 receptor: an enigma with multiple variations Am J Physiol Endocrinol Metab 2000 278 E357 374 10.1152/ajpendo.2000.278.3.E357 10710489

[B18] Gimonet V Bussieres L Medjebeur AA Gasser B Lelongt B Laborde K Nephrogenesis and angiotensin II receptor subtypes gene expression in the fetal lamb Am J Physiol 1998 274 F1062 1069 10.1152/ajprenal.1998.274.6.F1062 9841497

[B19] Wolf G Butzmann U Wenzel UO The renin-angiotensin system and progression of renal disease: from hemodynamics to cell biology Nephron Physiol 2003 93 P3 13 10.1159/000066656 12411725

[B20] Horiuchi M Akishita M Dzau VJ Recent progress in angiotensin II type 2 receptor research in the cardiovascular system Hypertension 1999 33 613 621 10.1161/01.hyp.33.2.613 10024316

[B21] Opie LH Sack MN Enhanced angiotensin II activity in heart failure: reevaluation of the counterregulatory hypothesis of receptor subtypes Circulation Res 2001 88 654 658 10.1161/hh0701.089175 11304486

[B22] Naito T Ma LJ Yang H Zuo Y Tang Y Han JY Kon V Fogo AB Angiotensin type 2 receptor actions contribute to angiotensin type 1 receptor blocker effects on kidney fibrosis Am J Physiol Renal Physiol 2010 298 F683 691 10.1152/ajprenal.00503.2009 PMC2838584 20042458

[B23] Regitz-Zagrosek V Friedel N Heymann A Bauer P Neuss M Rolfs A Steffen C Hildebrandt A Hetzer R Fleck E Regulation, chamber localization, and subtype distribution of angiotensin II receptors in human hearts Circulation 1995 91 1461 1471 10.1161/01.cir.91.5.1461 7867188

[B24] Asano K Dutcher DL Port JD Minobe WA Tremmel KD Roden RL Bohlmeyer TJ Bush EW Jenkin MJ Abraham WT Raynolds MV Zisman LS Perryman MB Bristow MR Selective downregulation of the angiotensin II AT1-receptor subtype in failing human ventricular myocardium Circulation 1997 95 1193 1200 10.1161/01.cir.95.5.1193 9054849

[B25] Ruiz-Ortega M Lorenzo O Ruperez M Blanco J Egido J Systemic infusion of angiotensin II into normal rats activates nuclear factor-kappaB and AP-1 in the kidney: role of AT(1) and AT(2) receptors Am J Pathol 2001 158 1743 1756 10.1016/s0002-9440(10)64130-2 PMC1891960 11337372

[B26] Wolf G Wenzel U Burns KD Harris RC Stahl RA Thaiss F Angiotensin II activates nuclear transcription factor-kappaB through AT1 and AT2 receptors Kidney Int 2002 61 1986 1995 10.1046/j.1523-1755.2002.00365.x 12028439

[B27] Cao Z Bonnet F Candido R Nesteroff SP Burns WC Kawachi H Shimizu F Carey RM De Gasparo M Cooper ME Angiotensin type 2 receptor antagonism confers renal protection in a rat model of progressive renal injury J Am Soc Nephrol 2002 13 1773 1787 10.1097/01.asn.0000019409.17099.33 12089373

[B28] Wolf G Wenzel U Assmann KJ Stahl RA Renal expression of aminopeptidase A in rats with two-kidney, one-clip hypertension Nephrol Dial Transplant 2000 15 1935 1942 10.1093/ndt/15.12.1935 11096137

[B29] Handa RK Krebs LT Harding JW Handa SE Angiotensin IV AT4-receptor system in the rat kidney Am J Physiol 1998 274 F290 299 10.1152/ajprenal.1998.274.2.F290 9486224

[B30] Albiston AL McDowall SG Matsacos D Sim P Clune E Mustafa T Lee J Mendelsohn FA Simpson RJ Connolly LM Chai SY Evidence that the angiotensin IV (AT(4)) receptor is the enzyme insulin-regulated aminopeptidase J Biol Chem 2001 276 48623 48626 10.1074/jbc.C100512200 11707427

[B31] Gesualdo L Ranieri E Monno R Rossiello MR Colucci M Semeraro N Grandaliano G Schena FP Ursi M Cerullo G Angiotensin IV stimulates plasminogen activator inhibitor-1 expression in proximal tubular epithelial cells Kidney Int 1999 56 461 470 10.1046/j.1523-1755.1999.00578.x 10432384

[B32] Ma LJ Fogo AB PAI-1 and kidney fibrosis Front Biosci 2009 14 2028 2041 10.2741/3361 PMC4848749 19273183

[B33] Hill-Kapturczak N Kapturczak MH Block ER Patel JM Malinski T Madsen KM Tisher CC Angiotensin II-stimulated nitric oxide release from porcine pulmonary endothelium is mediated by angiotensin IV J Am Soc Nephrol 1999 10 481 491 10.1681/ASN.V103481 10073598

[B34] Chen JK Zimpelmann J Harris RC Burns KD Angiotensin IV induces tyrosine phosphorylation of focal adhesion kinase and paxillin in proximal tubule cells Am J Physiol Renal Physiol 2001 280 F980 988 10.1152/ajprenal.2001.280.6.F980 11352837

[B35] Wong YC Sim MK Lee KO Des-aspartate-angiotensin-I and angiotensin IV improve glucose tolerance and insulin signalling in diet-induced hyperglycaemic mice Biochem Pharmacol 2011 10.1016/j.bcp.2011.07.080 21803028

[B36] Siebelmann M Wensing J Verspohl EJ The impact of ANG II and IV on INS-1 cells and on blood glucose and plasma insulin J Recept Signal Transduct Res 2010 30 234 245 10.3109/10799893.2010.487491 20524779

[B37] Gard PR Cognitive-enhancing effects of angiotensin IV BMC Neurosci 2008 9 Suppl 2 S15 10.1186/1471-2202-9-S2-S15 PMC2604899 19090988

[B38] Kohara K Brosnihan KB Chappell MC Khosla MC Ferrario CM Angiotensin-(1-7). A member of circulating angiotensin peptides Hypertension 1991 17 131 138 10.1161/01.hyp.17.2.131 1846840

[B39] Freeman EJ Chisolm GM Ferrario CM Tallant EA Angiotensin-(1-7) inhibits vascular smooth muscle cell growth Hypertension 1996 28 104 108 10.1161/01.hyp.28.1.104 8675248

[B40] Strawn WB Ferrario CM Tallant EA Angiotensin-(1-7) reduces smooth muscle growth after vascular injury Hypertension 1999 33 207 211 10.1161/01.hyp.33.1.207 9931106

[B41] Benter IF Ferrario CM Morris M Diz DI Antihypertensive actions of angiotensin-(1-7) in spontaneously hypertensive rats Am J Physiol 1995 269 H313 319 10.1152/ajpheart.1995.269.1.H313 7631863

[B42] Clark MA Diz DI Tallant EA Angiotensin-(1-7) downregulates the angiotensin II type 1 receptor in vascular smooth muscle cells Hypertension 2001 37 1141 1146 10.1161/01.hyp.37.4.1141 11304516

[B43] Santos RA Simoes e Silva AC Maric C Silva DM Machado RP de Buhr I Heringer-Walther S Pinheiro SV Lopes MT Bader M Mendes EP Lemos VS Campagnole-Santos MJ Schultheiss HP Speth R Walther T Angiotensin-(1-7) is an endogenous ligand for the G protein-coupled receptor Mas Proc Natl Acad Sci USA 2003 100 8258 8263 10.1073/pnas.1432869100 PMC166216 12829792

[B44] Walther T Wessel N Kang N Sander A Tschope C Malberg H Bader M Voss A Altered heart rate and blood pressure variability in mice lacking the Mas protooncogene Braz J Med Biol Res 2000 33 1 9 10.1590/s0100-879x2000000100001 10625868

[B45] Peiro C Vallejo S Gembardt F Azcutia V Heringer-Walther S Rodriguez-Manas L Schultheiss HP Sanchez-Ferrer CF Walther T Endothelial dysfunction through genetic deletion or inhibition of the G protein-coupled receptor Mas: a new target to improve endothelial function J Hypertens 2007 25 2421 2425 10.1097/HJH.0b013e3282f0143c 17984663

[B46] Xu P Santos RA Bader M Alenina N Alterations in gene expression in the testis of angiotensin-(1-7)-receptor Mas-deficient mice Regul Pept 2007 138 51 55 10.1016/j.regpep.2006.11.017 17196677

[B47] Crackower MA Sarao R Oudit GY Yagil C Kozieradzki I Scanga SE Oliveira-dos-Santos AJ da Costa J Zhang L Pei Y Scholey J Ferrario CM Manoukian AS Chappell MC Backx PH Yagil Y Penninger JM Angiotensin-converting enzyme 2 is an essential regulator of heart function Nature 2002 417 822 828 10.1038/nature00786 12075344

[B48] Vickers C Hales P Kaushik V Dick L Gavin J Tang J Godbout K Parsons T Baronas E Hsieh F Acton S Patane M Nichols A Tummino P Hydrolysis of biological peptides by human angiotensin-converting enzyme-related carboxypeptidase J Biol Chem 2002 277 14838 14843 10.1074/jbc.M200581200 11815627

[B49] Tikellis C Johnston CI Forbes JM Burns WC Burrell LM Risvanis J Cooper ME Characterization of renal angiotensin-converting enzyme 2 in diabetic nephropathy Hypertension 2003 41 392 397 10.1161/01.HYP.0000060689.38912.CB 12623933

[B50] Ferreira AJ Santos RA Bradford CN Mecca AP Sumners C Katovich MJ Raizada MK Therapeutic implications of the vasoprotective axis of the renin-angiotensin system in cardiovascular diseases Hypertension 2010 55 207 213 10.1161/HYPERTENSIONAHA.109.140145 PMC2826213 20038757

[B51] Perret-Guillaume C Joly L Jankowski P Benetos A Benefits of the RAS blockade: clinical evidence before the ONTARGET study J Hypertens Suppl 2009 27 S3 7 10.1097/01.hjh.0000354511.14086.f1 19491620

[B52] Pereira RM dos Santos RA da Costa Dias FL Teixeira MM Simoes e Silva AC Renin-angiotensin system in the pathogenesis of liver fibrosis World J Gastroenterol 2009 15 2579 2586 10.3748/wjg.15.2579 PMC2691487 19496186

[B53] Tharaux PL Chatziantoniou C Fakhouri F Dussaule JC Angiotensin II activates collagen I gene through a mechanism involving the MAP/ER kinase pathway Hypertension 2000 36 330 336 10.1161/01.hyp.36.3.330 10988260

[B54] Yaghini FA Song CY Lavrentyev EN Ghafoor HU Fang XR Estes AM Campbell WB Malik KU Angiotensin II-induced vascular smooth muscle cell migration and growth are mediated by cytochrome P450 1B1-dependent superoxide generation Hypertension 2010 55 1461 1467 10.1161/HYPERTENSIONAHA.110.150029 PMC2916227 20439821

[B55] Xie Z Singh M Singh K ERK1/2 and JNKs, but not p38 kinase, are involved in reactive oxygen species-mediated induction of osteopontin gene expression by angiotensin II and interleukin-1beta in adult rat cardiac fibroblasts J Cell Physiol 2004 198 399 407 10.1002/jcp.10419 14755545

[B56] Li L Fan D Wang C Wang JY Cui XB Wu D Zhou Y Wu LL Angiotensin II increases periostin expression via Ras/p38 MAPK/CREB and ERK1/2/TGF-β1 pathways in cardiac fibroblasts Cardiovasc Res 2011 91 80 89 10.1093/cvr/cvr067 21367774

[B57] Yang F Chung AC Huang XR Lan HY Angiotensin II induces connective tissue growth factor and collagen I expression via transforming growth factor-beta-dependent and -independent Smad pathways: the role of Smad3 Hypertension 2009 54 877 884 10.1161/HYPERTENSIONAHA.109.136531 19667256

[B58] Zeng W Chen W Leng X He JG Ma H Chronic angiotensin-(1-7) administration improves vascular remodeling after angioplasty through the regulation of the TGF-beta/Smad signaling pathway in rabbits Biochem Biophys Res Commun 2009 389 138 144 10.1016/j.bbrc.2009.08.112 19715669

[B59] Carvajal G Rodriguez-Vita J Rodrigues-Diez R Sanchez-Lopez E Ruperez M Cartier C Esteban V Ortiz A Egido J Mezzano SA Ruiz-Ortega M Angiotensin II activates the Smad pathway during epithelial mesenchymal transdifferentiation Kidney Int 2008 74 585 595 10.1038/ki.2008.213 18509316

[B60] Rodriguez-Vita J Sanchez-Lopez E Esteban V Ruperez M Egido J Ruiz-Ortega M Angiotensin II activates the Smad pathway in vascular smooth muscle cells by a transforming growth factor-beta-independent mechanism Circulation 2005 111 2509 2517 10.1161/01.CIR.0000165133.84978.E2 15883213

[B61] Liou JY Hong HJ Sung LC Chao HH Chen PY Cheng TH Chan P Liu JC Nicorandil Inhibits Angiotensin-II-Induced Proliferation of Cultured Rat Cardiac Fibroblasts Pharmacology 2011 87 144 151 10.1159/000323555 21346392

[B62] Wilmer WA Hebert LA Lewis EJ Rohde RD Whittier F Cattran D Levey AS Lewis JB Spitalewitz S Blumenthal S Bain RP Remission of nephrotic syndrome in type 1 diabetes: long-term follow-up of patients in the Captopril Study Am J Kidney Dis 1999 34 308 314 10.1016/s0272-6386(99)70360-4 10430979

[B63] Group TG Randomised placebo-controlled trial of effect of ramipril on decline in glomerular filtration rate and risk of terminal renal failure in proteinuric, non-diabetic nephropathy. The GISEN Group (Gruppo Italiano di Studi Epidemiologici in Nefrologia) Lancet 1997 349 1857 1863 9217756

[B64] Maschio G Alberti D Locatelli F Mann JF Motolese M Ponticelli C Ritz E Janin G Zucchelli P Angiotensin-converting enzyme inhibitors and kidney protection: the AIPRI trial. The ACE Inhibition in Progressive Renal Insufficiency (AIPRI) Study Group J Cardiovasc Pharmacol 1999 33 Suppl 1 S16 20 10.1097/00005344-199900001-00004 10028949

[B65] Ruggenenti P Perna A Gherardi G Gaspari F Benini R Remuzzi G Renal function and requirement for dialysis in chronic nephropathy patients on long-term ramipril: REIN follow-up trial. Gruppo Italiano di Studi Epidemiologici in Nefrologia (GISEN). Ramipril Efficacy in Nephropathy Lancet 1998 352 1252 1256 10.1016/s0140-6736(98)04433-x 9788454

[B66] Tyralla K Adamczak M Benz K Campean V Gross ML Hilgers KF Ritz E Amann K High-dose enalapril treatment reverses myocardial fibrosis in experimental uremic cardiomyopathy PLoS ONE 2011 6 e15287 10.1371/journal.pone.0015287 PMC3029304 21298056

[B67] Gross O Schulze-Lohoff E Koepke ML Beirowski B Addicks K Bloch W Smyth N Weber M Antifibrotic, nephroprotective potential of ACE inhibitor vs AT1 antagonist in a murine model of renal fibrosis Nephrol Dial Transplant 2004 19 1716 1723 10.1093/ndt/gfh219 15128880

[B68] Tang SC Leung JC Chan LY Eddy AA Lai KN Angiotensin converting enzyme inhibitor but not angiotensin receptor blockade or statin ameliorates murine adriamycin nephropathy Kidney Int 2008 73 288 299 10.1038/sj.ki.5002674 18033243

[B69] Li Q Wang Y Sun SZ Tian YJ Liu MH Effects of benazepril on cardiac fibrosis in STZ-induced diabetic rats Acta Cardiol 2010 65 431 439 10.2143/AC.65.4.2053902 20821936

[B70] Imig JD Navar GL Zou LX O'Reilly KC Allen PL Kaysen JH Hammond TG Navar LG Renal endosomes contain angiotensin peptides, converting enzyme, and AT(1A) receptors Am J Physiol 1999 277 F303 311 10.1152/ajprenal.1999.277.2.F303 10444586

[B71] Lenfant M Wdzieczak-Bakala J Guittet E Prome JC Sotty D Frindel E Inhibitor of hematopoietic pluripotent stem cell proliferation: purification and determination of its structure Proc Natl Acad Sci USA 1989 86 779 782 10.1073/pnas.86.3.779 PMC286560 2915977

[B72] Grillon C Rieger K Bakala J Schott D Morgat JL Hannappel E Voelter W Lenfant M Involvement of thymosin beta 4 and endoproteinase Asp-N in the biosynthesis of the tetrapeptide AcSerAspLysPro a regulator of the hematopoietic system FEBS Lett 1990 274 30 34 10.1016/0014-5793(90)81322-f 2253778

[B73] Liu JM Garcia-Alvarez MC Bignon J Kusinski M Kuzdak K Riches A Wdzieczak-Bakala J Overexpression of the natural tetrapeptide acetyl-N-ser-asp-lys-pro derived from thymosin beta4 in neoplastic diseases Ann NY Acad Sci 2010 1194 53 59 10.1111/j.1749-6632.2010.05488.x 20536450

[B74] Cavasin MA Rhaleb NE Yang XP Carretero OA Prolyl oligopeptidase is involved in release of the antifibrotic peptide Ac-SDKP Hypertension 2004 43 1140 1145 10.1161/01.HYP.0000126172.01673.84 PMC4677773 15037553

[B75] Huff T Muller CS Otto AM Netzker R Hannappel E beta-Thymosins, small acidic peptides with multiple functions Int J Biochem Cell Biol 2001 33 205 220 10.1016/s1357-2725(00)00087-x 11311852

[B76] Hannappel E Thymosin beta4 and its posttranslational modifications Ann NY Acad Sci 2010 1194 27 35 10.1111/j.1749-6632.2010.05485.x 20536447

[B77] Bock-Marquette I Saxena A White MD Dimaio JM Srivastava D Thymosin beta4 activates integrin-linked kinase and promotes cardiac cell migration, survival and cardiac repair Nature 2004 432 466 472 10.1038/nature03000 15565145

[B78] Smart N Risebro CA Melville AA Moses K Schwartz RJ Chien KR Riley PR Thymosin beta4 induces adult epicardial progenitor mobilization and neovascularization Nature 2007 445 177 182 10.1038/nature05383 17108969

[B79] Wdzieczak-Bakala J Fache MP Lenfant M Frindel E Sainteny F AcSDKP, an inhibitor of CFU-S proliferation, is synthesized in mice under steady-state conditions and secreted by bone marrow in long-term culture Leukemia 1990 4 235 237 2314120

[B80] Azizi M Rousseau A Ezan E Guyene TT Michelet S Grognet JM Lenfant M Corvol P Menard J Acute angiotensin-converting enzyme inhibition increases the plasma level of the natural stem cell regulator N-acetyl-seryl-aspartyl-lysyl-proline J Clin Invest 1996 97 839 844 10.1172/JCI118484 PMC507123 8609242

[B81] Kanasaki K Haneda M Sugimoto T Shibuya K Isono M Isshiki K Araki S Uzu T Kashiwagi A Koya D N-acetyl-seryl-aspartyl-lysyl-proline inhibits DNA synthesis in human mesangial cells via up-regulation of cell cycle modulators Biochem Biophys Res Commun 2006 342 758 765 10.1016/j.bbrc.2006.02.019 16497271

[B82] Iwamoto N Xano HJ Yoshioka T Shiraga H Nitta K Muraki T Ito K Acetyl-seryl-aspartyl-lysyl-proline is a novel natural cell cycle regulator of renal cells Life Sci 2000 66 PL221 226 10.1016/s0024-3205(00)00460-4 11210724

[B83] Rhaleb NE Peng H Harding P Tayeh M LaPointe MC Carretero OA Effect of N-acetyl-seryl-aspartyl-lysyl-proline on DNA and collagen synthesis in rat cardiac fibroblasts Hypertension 2001 37 827 832 10.1161/01.hyp.37.3.827 PMC6824426 11244003

[B84] Rhaleb NE Peng H Yang XP Liu YH Mehta D Ezan E Carretero OA Long-term effect of N-acetyl-seryl-aspartyl-lysyl-proline on left ventricular collagen deposition in rats with 2-kidney, 1-clip hypertension Circulation 2001 103 3136 3141 10.1161/01.cir.103.25.3136 PMC4679287 11425781

[B85] Peng H Carretero OA Raij L Yang F Kapke A Rhaleb NE Antifibrotic effects of N-acetyl-seryl-aspartyl-lysyl-proline on the heart and kidney in aldosterone-salt hypertensive rats Hypertension 2001 37 794 800 10.1161/01.hyp.37.2.794 PMC6824419 11230375

[B86] Pokharel S Rasoul S Roks AJ van Leeuwen RE van Luyn MJ Deelman LE Smits JF Carretero O van Gilst WH Pinto YM N-acetyl-Ser-Asp-Lys-Pro inhibits phosphorylation of Smad2 in cardiac fibroblasts Hypertension 2002 40 155 161 10.1161/01.hyp.0000025880.56816.fa 12154106

[B87] Kanasaki K Koya D Sugimoto T Isono M Kashiwagi A Haneda M N-Acetyl-seryl-aspartyl-lysyl-proline inhibits TGF-beta-mediated plasminogen activator inhibitor-1 expression via inhibition of Smad pathway in human mesangial cells J Am Soc Nephrol 2003 14 863 872 10.1097/01.asn.0000057544.95569.ec 12660320

[B88] Border WA Noble NA Transforming growth factor beta in tissue fibrosis New Engl J Med 1994 331 1286 1292 10.1056/NEJM199411103311907 7935686

[B89] Miyazono K TGF-beta signaling by Smad proteins Cytokine Growth Factor Rev 2000 11 15 22 10.1016/s1359-6101(99)00025-8 10708949

[B90] Wrana JL Attisano L Carcamo J Zentella A Doody J Laiho M Wang XF Massague J TGF beta signals through a heteromeric protein kinase receptor complex Cell 1992 71 1003 1014 10.1016/0092-8674(92)90395-s 1333888

[B91] Xiao Z Liu X Lodish HF Importin beta mediates nuclear translocation of Smad 3 J Biol Chem 2000 275 23425 23428 10.1074/jbc.C000345200 10846168

[B92] Kurisaki A Kose S Yoneda Y Heldin CH Moustakas A Transforming growth factor-beta induces nuclear import of Smad3 in an importin-beta1 and Ran-dependent manner Mol Biol Cell 2001 12 1079 1091 10.1091/mbc.12.4.1079 PMC32288 11294908

[B93] Nakao A Afrakhte M Moren A Nakayama T Christian JL Heuchel R Itoh S Kawabata M Heldin NE Heldin CH ten Dijke P Identification of Smad7, a TGFbeta-inducible antagonist of TGF-beta signalling Nature 1997 389 631 635 10.1038/39369 9335507

[B94] Ebisawa T Fukuchi M Murakami G Chiba T Tanaka K Imamura T Miyazono K Smurf1 interacts with transforming growth factor-beta type I receptor through Smad7 and induces receptor degradation J Biol Chem 2001 276 12477 12480 10.1074/jbc.C100008200 11278251

[B95] Suzuki C Murakami G Fukuchi M Shimanuki T Shikauchi Y Imamura T Miyazono K Smurf1 regulates the inhibitory activity of Smad7 by targeting Smad7 to the plasma membrane J Biol Chem 2002 277 39919 39925 10.1074/jbc.M201901200 12151385

[B96] Gronroos E Hellman U Heldin CH Ericsson J Control of Smad7 stability by competition between acetylation and ubiquitination Mol Cell 2002 10 483 493 10.1016/s1097-2765(02)00639-1 12408818

[B97] Omata M Taniguchi H Koya D Kanasaki K Sho R Kato Y Kojima R Haneda M Inomata N N-acetyl-seryl-aspartyl-lysyl-proline ameliorates the progression of renal dysfunction and fibrosis in WKY rats with established anti-glomerular basement membrane nephritis J Am Soc Nephrol 2006 17 674 685 10.1681/ASN.2005040385 16452498

[B98] Lin CX Rhaleb NE Yang XP Liao TD D'Ambrosio MA Carretero OA Prevention of aortic fibrosis by N-acetyl-seryl-aspartyl-lysyl-proline in angiotensin II-induced hypertension Am J Physiol Heart Circ Physiol 2008 295 H1253 H1261 10.1152/ajpheart.00481.2008 PMC2544498 18641275

[B99] Shah SA Potter MW Callery MP Ubiquitin proteasome pathway: implications and advances in cancer therapy Surg Oncol 2001 10 43 52 10.1016/s0960-7404(01)00018-4 11719028

[B100] Bernstein KE Shen XZ Gonzalez-Villalobos RA Billet S Okwan-Duodu D Ong FS Fuchs S Different *in vivo *functions of the two catalytic domains of angiotensin-converting enzyme (ACE) Curr Opin Pharmacol 2011 11 105 111 10.1016/j.coph.2010.11.001 PMC3075415 21130035

[B101] Wei L Alhenc-Gelas F Corvol P Clauser E The two homologous domains of human angiotensin I-converting enzyme are both catalytically active J Biol Chem 1991 266 9002 9008 1851160

[B102] Rousseau A Michaud A Chauvet MT Lenfant M Corvol P The hemoregulatory peptide N-acetyl-Ser-Asp-Lys-Pro is a natural and specific substrate of the N-terminal active site of human angiotensin-converting enzyme J Biol Chem 1995 270 3656 3661 10.1074/jbc.270.8.3656 7876104

[B103] Zisman LS Inhibiting tissue angiotensin-converting enzyme: a pound of flesh without the blood? Circulation 1998 98 2788 2790 10.1161/01.cir.98.25.2788 9860776

[B104] Li P Xiao HD Xu J Ong FS Kwon M Roman J Gal A Bernstein KE Fuchs S Angiotensin-converting enzyme N-terminal inactivation alleviates bleomycin-induced lung injury Am J Pathol 2010 177 1113 1121 10.2353/ajpath.2010.081127 PMC2928946 20651228

[B105] Stephan J Melaine N Ezan E Hakovirta H Maddocks S Toppari J Garnier D Wdzieczak-Bakala J Jegou B Source, catabolism and role of the tetrapeptide N-acetyl-ser-asp-lys-Pro within the testis J Cell Sci 2000 113 113 121 10.1242/jcs.113.1.113 10591630

[B106] Fuchs S Xiao HD Cole JM Adams JW Frenzel K Michaud A Zhao H Keshelava G Capecchi MR Corvol P Bernstein KE Role of the N-terminal catalytic domain of angiotensin-converting enzyme investigated by targeted inactivation in mice J Biol Chem 2004 279 15946 15953 10.1074/jbc.M400149200 14757757

[B107] Junot C Gonzales MF Ezan E Cotton J Vazeux G Michaud A Azizi M Vassiliou S Yiotakis A Corvol P Dive V RXP 407, a selective inhibitor of the N-domain of angiotensin I-converting enzyme, blocks *in vivo *the degradation of hemoregulatory peptide acetyl-Ser-Asp-Lys-Pro with no effect on angiotensin I hydrolysis J Pharmacol Exp Ther 2001 297 606 611 11303049

[B108] Vazeux G Cotton J Cuniasse P Dive V Potency and selectivity of RXP407 on human, rat, and mouse angiotensin-converting enzyme Biochem Pharmacol 2001 61 835 841 10.1016/s0006-2952(01)00550-0 11274969

[B109] Kroger WL Douglas RG O'Neill HG Dive V Sturrock ED Investigating the domain specificity of phosphinic inhibitors RXPA380 and RXP407 in angiotensin-converting enzyme Biochemistry 2009 48 8405 8412 10.1021/bi9011226 19658433

[B110] Anthony CS Corradi HR Schwager SL Redelinghuys P Georgiadis D Dive V Acharya KR Sturrock ED The N domain of human angiotensin-I-converting enzyme: the role of N-glycosylation and the crystal structure in complex with an N domain-specific phosphinic inhibitor, RXP407 J Biol Chem 2010 285 35685 35693 10.1074/jbc.M110.167866 PMC2975193 20826823

[B111] Bomback AS Klemmer PJ The incidence and implications of aldosterone breakthrough Nat Clin Pract Nephrol 2007 3 486 492 10.1038/ncpneph0575 17717561

[B112] Brenner BM Cooper ME de Zeeuw D Keane WF Mitch WE Parving HH Remuzzi G Snapinn SM Zhang Z Shahinfar S Effects of losartan on renal and cardiovascular outcomes in patients with type 2 diabetes and nephropathy New Engl J Med 2001 345 861 869 10.1056/NEJMoa011161 11565518

[B113] Erdos EG Tan F Skidgel RA Angiotensin I-converting enzyme inhibitors are allosteric enhancers of kinin B1 and B2 receptor function Hypertension 2010 55 214 220 10.1161/HYPERTENSIONAHA.109.144600 PMC2814794 20065150

[B114] Schanstra JP Neau E Drogoz P Arevalo Gomez MA Lopez Novoa JM Calise D Pecher C Bader M Girolami JP Bascands JL *In vivo *bradykinin B2 receptor activation reduces renal fibrosis J Clin Invest 2002 110 371 379 10.1172/JCI15493 PMC151090 12163456

[B115] Kakoki M Takahashi N Jennette JC Smithies O Diabetic nephropathy is markedly enhanced in mice lacking the bradykinin B2 receptor Proc Natl Acad Sci USA 2004 101 13302 13305 10.1073/pnas.0405449101 PMC516527 15326315

[B116] Schanstra JP Duchene J Desmond L Neau E Calise D Estaque S Girolami JP Bascands JL The protective effect of angiotensin converting enzyme inhibition in experimental renal fibrosis in mice is not mediated by bradykinin B2 receptor activation Thromb Haemost 2003 89 735 740 12669129

[B117] Pawluczyk IZ Patel SR Harris KP The role of bradykinin in the antifibrotic actions of perindoprilat on human mesangial cells Kidney Int 2004 65 1240 1251 10.1111/j.1523-1755.2004.00494.x 15086463

[B118] Mogensen CE Neldam S Tikkanen I Oren S Viskoper R Watts RW Cooper ME Randomised controlled trial of dual blockade of renin-angiotensin system in patients with hypertension, microalbuminuria, and non-insulin dependent diabetes: the candesartan and lisinopril microalbuminuria (CALM) study Br Med J 2000 321 1440 1444 10.1136/bmj.321.7274.1440 PMC27545 11110735

[B119] Russo D Minutolo R Pisani A Esposito R Signoriello G Andreucci M Balletta MM Coadministration of losartan and enalapril exerts additive antiproteinuric effect in IgA nephropathy Am J Kidney Dis 2001 38 18 25 10.1053/ajkd.2001.25176 11431176

[B120] Kunz R Friedrich C Wolbers M Mann JF Meta-analysis: effect of monotherapy and combination therapy with inhibitors of the renin angiotensin system on proteinuria in renal disease Ann Intern Med 2008 148 30 48 10.7326/0003-4819-148-1-200801010-00190 17984482

[B121] Granger CB McMurray JJ Yusuf S Held P Michelson EL Olofsson B Ostergren J Pfeffer MA Swedberg K Effects of candesartan in patients with chronic heart failure and reduced left-ventricular systolic function intolerant to angiotensin-converting-enzyme inhibitors: the CHARM-Alternative trial Lancet 2003 362 772 776 10.1016/S0140-6736(03)14284-5 13678870

[B122] McMurray JJ Ostergren J Swedberg K Granger CB Held P Michelson EL Olofsson B Yusuf S Pfeffer MA Effects of candesartan in patients with chronic heart failure and reduced left-ventricular systolic function taking angiotensin-converting-enzyme inhibitors: the CHARM-Added trial Lancet 2003 362 767 771 10.1016/S0140-6736(03)14283-3 13678869

[B123] Yusuf S Pfeffer MA Swedberg K Granger CB Held P McMurray JJ Michelson EL Olofsson B Ostergren J Effects of candesartan in patients with chronic heart failure and preserved left-ventricular ejection fraction: the CHARM-Preserved Trial Lancet 2003 362 777 781 10.1016/S0140-6736(03)14285-7 13678871

[B124] Mann JF Schmieder RE McQueen M Dyal L Schumacher H Pogue J Wang X Maggioni A Budaj A Chaithiraphan S Dickstein K Keltai M Metsärinne K Oto A Parkhomenko A Piegas LS Svendsen TL Teo KK Yusuf S ONTARGET investigators Renal outcomes with telmisartan, ramipril, or both, in people at high vascular risk (the ONTARGET study): a multicentre, randomised, double-blind, controlled trial Lancet 2008 372 547 553 10.1016/S0140-6736(08)61236-2 18707986

[B125] Mauer M Zinman B Gardiner R Suissa S Sinaiko A Strand T Drummond K Donnelly S Goodyer P Gubler MC Klein R Renal and retinal effects of enalapril and losartan in type 1 diabetes New Engl J Med 2009 361 40 51 10.1056/NEJMoa0808400 PMC2978030 19571282

[B126] Metsarinne KP Helin KH Saijonmaa O Stewen P Sirvio ML Fyhrquist FY Tissue-specific regulation of angiotensin-converting enzyme by angiotensin II and losartan in the rat Blood Press 1996 5 363 370 10.3109/08037059609078076 8973755

[B127] Pradelles P Frobert Y Creminon C Ivonine H Frindel E Distribution of a negative regulator of haematopoietic stem cell proliferation (AcSDKP) and thymosin beta 4 in mouse tissues FEBS Lett 1991 289 171 175 10.1016/0014-5793(91)81062-d 1915845

[B128] Pradelles P Frobert Y Creminon C Liozon E Masse A Frindel E Negative regulator of pluripotent hematopoietic stem cell proliferation in human white blood cells and plasma as analysed by enzyme immunoassay Biochem Biophys Res Commun 1990 170 986 993 10.1016/0006-291x(90)90489-a 2202303

[B129] Bogden AE Carde P de Paillette ED Moreau JP Tubiana M Frindel E Amelioration of chemotherapy-induced toxicity by cotreatment with AcSDKP, a tetrapeptide inhibitor of hematopoietic stem cell proliferation Ann N Y Acad Sci 1991 628 126 139 10.1111/j.1749-6632.1991.tb17230.x 1648882

[B130] Grillon C Bonnet D Mary JY Lenfant M Najman A Guigon M The tetrapeptide AcSerAspLysPro (Seraspenide), a hematopoietic inhibitor, may reduce the *in vitro *toxicity of 3'-azido-3'-deoxythymidine to human hematopoietic progenitors Stem Cells 1993 11 455 464 10.1002/stem.5530110513 8241956

[B131] Deeg HJ Seidel K Hong DS Yu C Huss R Schuening FG Ewel CH Storb R *In vivo *radioprotective effect of AcSDKP on canine myelopoiesis Ann Hematol 1997 74 117 122 10.1007/s002770050268 9111424

[B132] Watanabe T Brown GS Kelsey LS Yan Y Jackson JD Ewel C Kessinger A Talmadge JE *In vivo *protective effects of tetrapeptide AcSDKP, with or without granulocyte colony-stimulation factor, on murine progenitor cells after sublethal irradiation Exp Hematol 1996 24 713 721 8635527

[B133] Wierenga PK Brenner MK Konings AW Enhanced selectivity of hyperthermic purging of human progenitor cells using Goralatide, an inhibitor of cell cycle progression Bone Marrow Transplant 1998 21 73 78 10.1038/sj.bmt.1701045 9486498

[B134] Wierenga PK Konings AW Seraspenide (AcSDKP) mediated protection of hematopoietic stem cells in a hyperthermic purging protocol Prog Clin Biol Res 1994 389 189 195 7700902

[B135] Wierenga PK Setroikromo R Vellenga E Kampinga HH Purging of acute myeloid leukaemia cells from stem cell grafts by hyperthermia: enhancement of the therapeutic index by the tetrapeptide AcSDKP and the alkyl-lysophospholipid ET-18-OCH(3) Br J Haematol 2000 111 1145 1152 10.1046/j.1365-2141.2000.02469.x 11167754

[B136] Coutton C Guigon M Bohbot A Ferrani K Oberling F Photoprotection of normal human hematopoietic progenitors by the tetrapeptide N-AcSDKP Exp Hematol 1994 22 1076 1080 7925774

[B137] Coward WR Saini G Jenkins G The pathogenesis of idiopathic pulmonary fibrosis Ther Adv Respir Dis 2010 4 367 388 10.1177/1753465810379801 20952439

[B138] Rodriguez-Iturbe B Garcia Garcia G The role of tubulointerstitial inflammation in the progression of chronic renal failure Nephron Clin Pract 2010 116 c81 88 10.1159/000314656 20502043

[B139] Gieling RG Burt AD Mann DA Fibrosis and cirrhosis reversibility-molecular mechanisms Clin Liver Dis 2008 12 915 937, xi 10.1016/j.cld.2008.07.001 18984474

[B140] Dooley R Harvey BJ Thomas W The regulation of cell growth and survival by aldosterone Front Biosci 2011 16 440 457 10.2741/3697 21196180

[B141] Chen YW Liu BW Zhang YJ Chen YW Dong GF Ding XD Xu LM Pat B Fan JG Li DG Preservation of basal AcSDKP attenuates carbon tetrachloride-induced fibrosis in the rat liver J Hepatol 2010 53 528 536 10.1016/j.jhep.2010.03.027 20646773

[B142] Liu YH D'Ambrosio M Liao TD Peng H Rhaleb NE Sharma U Andre S Gabius HJ Carretero OA *N*-acetyl-seryl-aspartyl-lysyl-proline prevents cardiac remodeling and dysfunction induced by galectin-3, a mammalian adhesion/growth-regulatory lectin Am J Physiol Heart Circ Physiol 2009 296 H404 412 10.1152/ajpheart.00747.2008 PMC2643891 19098114

[B143] Sharma U Rhaleb NE Pokharel S Harding P Rasoul S Peng H Carretero OA Novel anti-inflammatory mechanisms of *N*-acetyl-Ser-Asp-Lys-Pro in hypertension-induced target organ damage Am J Physiol Heart Circ Physiol 2008 294 H1226 1232 10.1152/ajpheart.00305.2007 PMC6824420 18178715

[B144] Peng H Carretero OA Liao TD Peterson EL Rhaleb NE Role of N-acetyl-seryl-aspartyl-lysyl-proline in the antifibrotic and anti-inflammatory effects of the angiotensin-converting enzyme inhibitor captopril in hypertension Hypertension 2007 49 695 703 10.1161/01.HYP.0000258406.66954.4f PMC3257515 17283252

[B145] Yang F Yang XP Liu YH Xu J Cingolani O Rhaleb NE Carretero OA Ac-SDKP reverses inflammation and fibrosis in rats with heart failure after myocardial infarction Hypertension 2004 43 229 236 10.1161/01.HYP.0000107777.91185.89 PMC3259854 14691195

[B146] Wang M Liu R Jia X Mu S Xie R N-acetyl-seryl-aspartyl-lysyl-proline attenuates renal inflammation and tubulointerstitial fibrosis in rats Int J Mol Med 2010 26 795 801 10.3892/ijmm_00000527 21042772

[B147] Sun Y Yang F Yan J Li Q Wei Z Feng H Wang R Zhang L Zhang X New anti-fibrotic mechanisms of n-acetyl-seryl-aspartyl-lysyl-proline in silicon dioxide-induced silicosis Life Sci 2010 87 232 239 10.1016/j.lfs.2010.06.016 20624403

[B148] Peng H Carretero OA Peterson EL Rhaleb NE Ac-SDKP inhibits transforming growth factor-beta1-induced differentiation of human cardiac fibroblasts into myofibroblasts Am J Physiol Heart Circ Physiol 2010 298 H1357 1364 10.1152/ajpheart.00464.2009 PMC2867434 20154264

[B149] Liu JM Lawrence F Kovacevic M Bignon J Papadimitriou E Lallemand JY Katsoris P Potier P Fromes Y Wdzieczak-Bakala J The tetrapeptide AcSDKP, an inhibitor of primitive hematopoietic cell proliferation, induces angiogenesis *in vitro *and *in vivo* Blood 2003 101 3014 3020 10.1182/blood-2002-07-2315 12480715

[B150] Wang D Carretero OA Yang XY Rhaleb NE Liu YH Liao TD Yang XP N-acetyl-seryl-aspartyl-lysyl-proline stimulates angiogenesis *in vitro *and *in vivo* Am J Physiol Heart Circ Physiol 2004 287 H2099 2105 10.1152/ajpheart.00592.2004 PMC6824423 15256375

[B151] Fromes Y Liu JM Kovacevic M Bignon J Wdzieczak-Bakala J The tetrapeptide acetyl-serine-aspartyl-lysine-proline improves skin flap survival and accelerates wound healing Wound Repair Regen 2006 14 306 312 10.1111/j.1743-6109.2006.00125.x 16808809

[B152] Koutrafouri V Leondiadis L Avgoustakis K Livaniou E Czarnecki J Ithakissios DS Evangelatos GP Effect of thymosin peptides on the chick chorioallantoic membrane angiogenesis model Biochim Biophys Acta 2001 1568 60 66 10.1016/s0304-4165(01)00200-8 11731086

[B153] Philp D Huff T Gho YS Hannappel E Kleinman HK The actin binding site on thymosin beta4 promotes angiogenesis FASEB J 2003 17 2103 2105 10.1096/fj.03-0121fje 14500546

[B154] Malinda KM Goldstein AL Kleinman HK Thymosin beta 4 stimulates directional migration of human umbilical vein endothelial cells FASEB J 1997 11 474 481 10.1096/fasebj.11.6.9194528 9194528

[B155] Liu JM Bignon J Ilic V Briscoe C Lallemand JY Riches A Wdzieczak-Bakala J Evidence for an association of high levels of endogenous Acetyl-Ser-Asp-Lys-Pro, a potent mediator of angiogenesis, with acute myeloid leukemia development Leuk Lymphoma 2006 47 1915 1920 10.1080/10428190600688131 17065006

[B156] Liu JM Kusinski M Ilic V Bignon J Hajem N Komorowski J Kuzdak K Stepien H Wdzieczak-Bakala J Overexpression of the angiogenic tetrapeptide AcSDKP in human malignant tumors Anticancer Res 2008 28 2813 2817 19035315

[B157] Liu JM Gora-Tybor J Grzybowska-Izydorczyk O Bignon J Robak T Wdzieczak-Bakala J Elevated plasma levels of the angiogenic tetrapeptide acetyl-ser-asp-lys-pro are found in some patients with hematologic malignancies Leuk Lymphoma 2009 50 2096 2097 10.3109/10428190903331074 19863169

[B158] Folkman J Angiogenesis: an organizing principle for drug discovery? Nat Rev Drug Discov 2007 6 273 286 10.1038/nrd2115 17396134

[B159] Nyberg P Xie L Kalluri R Endogenous inhibitors of angiogenesis Cancer Res 2005 65 3967 3979 10.1158/0008-5472.CAN-04-2427 15899784

[B160] Zeisberg EM Tarnavski O Zeisberg M Dorfman AL McMullen JR Gustafsson E Chandraker A Yuan X Pu WT Roberts AB Neilson EG Sayegh MH Izumo S Kalluri R Endothelial-to-mesenchymal transition contributes to cardiac fibrosis Nat Med 2007 13 952 961 10.1038/nm1613 17660828

[B161] Zeisberg M Hanai J Sugimoto H Mammoto T Charytan D Strutz F Kalluri R BMP-7 counteracts TGF-beta1-induced epithelial-to-mesenchymal transition and reverses chronic renal injury Nat Med 2003 9 964 968 10.1038/nm888 12808448

[B162] Rieger KJ Saez-Servent N Papet MP Wdzieczak-Bakala J Morgat JL Thierry J Voelter W Lenfant M Involvement of human plasma angiotensin I-converting enzyme in the degradation of the haemoregulatory peptide N-acetyl-seryl-aspartyl-lysyl-proline Biochem J 1993 296 373 378 10.1042/bj2960373 PMC1137706 8257427

[B163] Ezan E Carde P Le Kerneau J Ardouin T Thomas F Isnard F Deschamps de Paillette E Grognet JM Pharmcokinetics in healthy volunteers and patients of NAc-SDKP (seraspenide), a negative regulator of hematopoiesis Drug Metab Dispos 1994 22 843 848 7895600

[B164] Robinson S Lenfant M Wdzieczak-Bakala J Riches A The molecular specificity of action of the tetrapeptide acetyl-N-Ser-Asp-Lys-Pro (AcSDKP) in the control of hematopoietic stem cell proliferation Stem Cells 1993 11 422 427 10.1002/stem.5530110509 8241953

[B165] Zhuo JL Carretero OA Peng H Li XC Regoli D Neugebauer W Rhaleb NE Characterization and localization of Ac-SDKP receptor binding sites using 125I-labeled Hpp-Aca-SDKP in rat cardiac fibroblasts Am J Physiol Heart Circ Physiol 2007 292 H984 993 10.1152/ajpheart.00776.2006 PMC2276842 17028162

